# In Situ Enzymatically Generated Photoswitchable Oxidase Mimetics and Their Application for Colorimetric Detection of Glucose Oxidase

**DOI:** 10.3390/molecules21070902

**Published:** 2016-07-09

**Authors:** Gen-Xia Cao, Xiu-Ming Wu, Yu-Ming Dong, Zai-Jun Li, Guang-Li Wang

**Affiliations:** The Key Laboratory of Food Colloids and Biotechnology, Ministry of Education, School of Chemical and Material Engineering, Jiangnan University, Wuxi 214122, China; 18206180706@163.com (G.-X.C.); wuxiuming821@163.com (X.-M.W.); dongym@jiangnan.edu.cn (Y.-M.D.); zaijunli@263.net (Z.-J.L.)

**Keywords:** glucose oxidase, photoswitchable oxidase mimetics, visible light, colorimetric sensor

## Abstract

In this study, a simple and amplified colorimetric assay is developed for the detection of the enzymatic activity of glucose oxidase (GOx) based on in situ formation of a photoswitchable oxidase mimetic of PO_4_^3−^-capped CdS quantum dots (QDs). GOx catalyzes the oxidation of 1-thio-β-d-glucose to give 1-thio-β-d-gluconic acid which spontaneously hydrolyzes to β-d-gluconic acid and H_2_S; the generated H_2_S instantly reacts with Cd^2+^ in the presence of Na_3_PO_4_ to give PO_4_^3−^-stabilized CdS QDs in situ. Under visible-light (λ ≥ 400 nm) stimulation, the PO_4_^3−^-capped CdS QDs are a new style of oxidase mimic derived by producing some active species, such as h^+^, ^•^OH, O_2_^•−^ and a little H_2_O_2_, which can oxidize the typical substrate (3,3,5,5-tetramethylbenzydine (TMB)) with a color change. Based on the GOx-triggered growth of the oxidase mimetics of PO_4_^3−^-capped CdS QDs in situ, we developed a simple and amplified colorimetric assay to probe the enzymatic activity of GOx. The proposed method allowed the detection of the enzymatic activity of GOx over the range from 25 μg/L to 50 mg/L with a low detection limit of 6.6 μg/L. We believe the PO_4_^3−^-capped CdS QDs generated in situ with photo-stimulated enzyme-mimicking activity may find wide potential applications in biosensors.

## 1. Introduction

Natural enzymes play an important role in biochemistry due to their high substrate specificity and high catalytic efficiency in catalyzing various meaningful reactions. Unfortunately, being a type of protein, natural enzymes suffer from some serious disadvantages: for example, (i) they can be easily denatured by environmental changes; (ii) they are prone to being digested by protease; and (iii) their preparation and purification are usually complex and expensive [1]. Accordingly, searching for artificial enzyme mimics with good stability and high catalytic capability is of great interest and urgently needed. Especially, the rapidly advancing field of nanotechnology supplies new possibilities for the development of enzyme mimics. Gao et al. [2] reported that Fe_3_O_4_ nanoparticles (NPs) possessed an intrinsic peroxidase-like activity in 2007, which opened the door for developing various nanoscale materials such as enzyme mimetics in the biochemical field. Since then, many manufactured nanomaterials have been found to possess peroxidase-like activity including noble metals [3,4], metal oxides [5], carbon materials [6,7,8,9] and so on. Owning to the prominent advantages of low cost, high stability, ease of storage, and tunability in catalytic activity, these nanomaterial-based mimicking enzymes are promising candidates for natural enzymes in biological and biomedical applications [10,11,12]. However, almost all of these nanomaterial-based peroxidase mimetics are ready-made ones; to construct exquisite nanoenzyme that can be integrated with biomolecules is becoming a significant field.

In this paper, we report an advanced method to detect the enzymatic activity of glucose oxidase (GOx) based on the GOx generation of S^2−^ anions followed by interaction with Cd^2+^/Na_3_PO_4_ to give PO_4_^3−^-capped CdS QDs in situ. Very interestingly, the PO_4_^3−^-capped CdS QDs were found to possess intrinsic oxidase-like activity under visible-light (λ ≥ 400 nm) stimulation, which could catalyze the oxidation of the typical substrate (3,3,5,5-tetramethylbenzydine (TMB)) with dissolved oxygen acting as the electron acceptor. Based on the GOx-triggered in situ growth of PO_4_^3−^-capped CdS QDs with intrinsic enzyme-like activity, we offered a novel approach to detect the enzymatic activity of GOx with efficient signal amplification. The mechanism of the catalytic reaction of the photoswitchable oxidase mimetics was also detected, proving that the generation of photo-generated holes (h^+^), hydroxyl radicals (^•^OH), hydrogen peroxide (H_2_O_2_) and especially superoxide anions (O_2_^•−^) comprised the reactive species. Compared to natural horseradish peroxidase (HRP) or the widely studied peroxidase mimetics based on nanomaterials, the photoswitchable oxidase mimetics of PO_4_^3−^-capped CdS QDs displayed several distinct advantages, such as the avoidance of damaging hydrogen peroxide, excellent enzyme-like activity, good stability even in harsh environments, and the easily triggered/controlled activity by visible light irradiation, etc., which fully demonstrated its great application foreground in promising biosensing and biotechnology. Considering that GOx is one of the most common enzymes (highly specific for β-d-glucose) that has been widely used in analytical chemistry due to its high turnover, specificity and stability [13,14], we believe that the protocol of coupling GOx with photoswitchable oxidase mimetics may find wide applications in biosensors.

## 2. Results and Discussion

### 2.1. The Photoswitchable Oxidase Mimetics of PO_4_^3−^-Capped CdS QDs Generated by GOx-Mediated Biocatalysis

**Figure 1:**
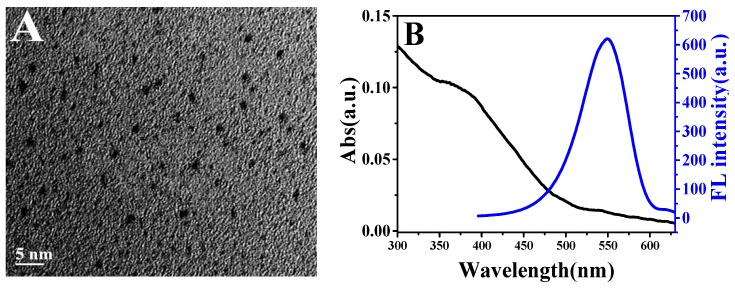
Characterization of the enzymatically generated CdS QDs. (**A**) HRTEM image and (**B**) the absorption (black line) and fluorescence (blue line) spectrum of PO_4_^3−^-capped CdS QDs produced by the enzymatic hydrolysis of 1-thio-β-d-glucose. PO_4_^3−^-capped CdS QDs were formed in the presence of GOx (20 mg/L), 1-thio-β-d-glucose (1 mM) and Cd^2+^ (1.5 mM), pH = 4.0.

GOx not only can enhance the oxidation of glucose, but it can also reinforce the oxidation of 1-thio-β-d-glucose to generate H_2_S [15]. The generated H_2_S could react immediately with cadmium cations and give CdS QDs. In addition, PO_4_^3−^ was used as a stabilizer for the formed CdS QDs to prevent particle aggregation [16]. The size of the CdS QDs catalyzed by GOx is about 2–3 nm (Figure 1A) as confirmed high resolution transmission electron microscopy (TEM). From the UV/vis spectrum of the formed PO_4_^3−^-capped CdS QDs (Figure 1B), we observe an absorption band from 300 to 480 nm and a shoulder peak at about 380 nm. The presence of this shoulder peak is explained by the 1S_h_–1S_e_ excitonic transition [17] characteristic of CdS NPs with a diameter of ~2–3 nm from the work of Peng et al. [18], which confirmed the data of the TEM analysis. The emission spectrum indicated (blue line) a well-shaped peak at 550 nm with an excitation of 290 nm, also demonstrating the typical fluorescence characteristics of QDs.

**Figure 2:**
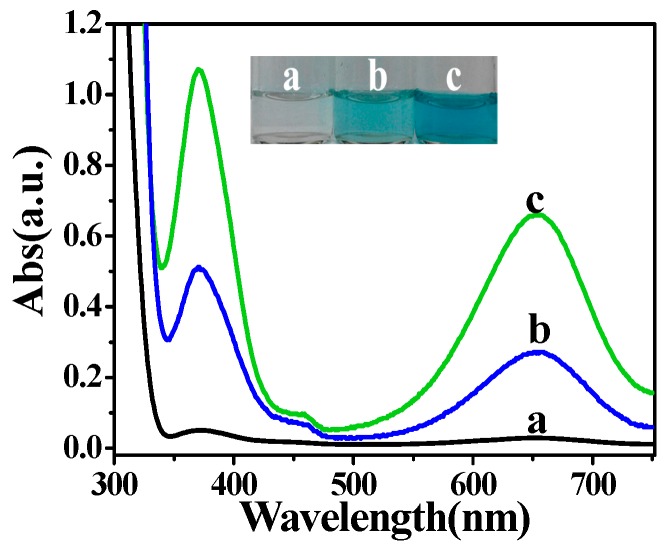
The UV/Vis spectra of (**a**) CdS + TMB; (**b**) CdS + TMB under visible-light irradiation; (**c**) PO_4_^3−^-capped CdS QDs + TMB under visible-light irradiation. CdS QDs were formed in the presence of GOx (20 mg/L), 1-thio-β-d-glucose (1 mM) and Cd^2+^ (1.5 mM), pH = 4.0. Irradiation time: 12 min. Inset image is the corresponding color of the above solutions.

Using TMB as the typical substrate of peroxidase/oxidase [19], the enzyme-mimicking activity of CdS QDs or PO_4_^3−^-capped CdS QDs under visible-light irradiation was investigated. As indicated in Figure 2, the formed CdS QDs could hardly induce the oxidation of TMB in the absence of visible light (line a). When the visible light (λ ≥ 400 nm) was introduced, two apparent absorption peaks at 370 nm and 652 nm appeared for CdS QDs (line b) and PO_4_^3−^-capped CdS QDs (line c), which were characteristic absorption peaks of oxidized TMB (oxTMB). However, neither Cd^2+^ nor S^2−^ alone could catalyze the oxidation of TMB under visible light (λ ≥ 400 nm) irradiation (Figure S1), which demonstrated that the enzyme-like catalytic activity was due to the photo-triggered CdS QDs. The bare CdS without surface stabilizers was easy to gather, resulting in the decrease of catalytic effect. Therefore, we imported a series of stabilizers to prevent its aggregation (Figure S2A). As reported, thioglycolic acid (TGA) was usually used to stabilize QDs. Because the covalent binding of thiol (-SH) and Cd^2+^ occurred, a protective layer with negative charges was formed on the surface of the QDs. This protective layer with electrostatic repulsion prevents direct contact between the quantum dots, leading to the formation of stable water-soluble nanoparticles. Similarly, Na_3_PO_4_, poly dimethyl diallyl ammonium chloride (PDDA) and chitosan (CS) could also stabilize QDs [16,20,21]. However, TGA may inhibit the catalytic effect in our system because of its reducibility. PDDA, CS, Na_3_PO_4_ could improve the catalytic effect with different degrees and the enhancement effect of Na_3_PO_4_ was the most obvious (Figure S2A). So we chose Na_3_PO_4_ as the stabilizer of CdS for obtaining enhanced catalytic activity. From Figure S2B, we found that the catalytic oxidation process leveled off in 12 min. Thus, 12 min was chosen as the illumination time.

Similar to natural enzymes, the relative catalytic activities of the PO_4_^3−^-capped CdS QDs were also influenced by the solution pH and temperature. It was found that the PO_4_^3−^-capped CdS QDs could retain relatively high activity at a wide range of pH (Figure S2C) and temperature (Figure S2D); the optimum reaction pH for PO_4_^3−^-capped CdS QDs was 3.0, while HRP (using H_2_O_2_ as an electron acceptor) achieved the highest catalytic activity at a pH of 5.0, and lower or higher pH both largely inhibited the catalytic activity. From Figure S2D, we can see that the PO_4_^3−^-capped CdS QDs keep a high catalytic activity in a wide range of temperatures from 20 to 90 °C, and the optimum reaction temperature was 43 °C, while HRP reached its maximum catalytic activity around 35 °C and showed a significant decrease in catalytic activity at lower or higher temperatures. These results demonstrated that PO_4_^3−^-capped CdS QDs as enzyme mimetics showed better stability and higher activity even under harsher conditions than that of HRP (using H_2_O_2_ as an electron acceptor).

We further examined the enzyme-like catalytic properties of PO_4_^3−^-capped CdS QDs under visible-light irradiation by steady-state kinetics. As shown in Figure S3, under the optimal conditions, typical Michaelis–Menten curves were obtained for PO_4_^3−^-capped CdS QDs with TMB as a substrate at a certain range of concentrations. The Michaelis-Menten constant (*K*_m_), an indicator of an enzyme’s affinity for its substrate, was obtained using Lineweaver–Burk plots (Figure S3 and Figure S4). The apparent kinetic parameters were calculated by the equation υ = *V*_max_ × [S]/(*K*_m_ + [S]), where υ is the initial enzymatic reaction rate, [S] is the concentration of the substrate, *V*_max_ is the maximum enzymatic reaction rate and *K*_m_ is the Michaelis-Menten constant. The apparent *K*_m_ value of the photo-activated PO_4_^3−^-capped CdS QDs with TMB as a substrate was 97.7 μM, which was much lower than that of HRP (*K*_m_ = 434 μM). In addition, the *V*_max_ was 42.86 nM·s^−1^. These results indicated that PO_4_^3−^-capped CdS QDs as a new kind of mimetic enzyme had a higher affinity for TMB than did HRP.

### 2.2. Mechanism of Photoswitchable Enzyme-Like Activity of PO_4_^3−^-Capped CdS QDs

With the purpose of verifying the catalytic mechanism, we primarily bubbled high-purity nitrogen into the catalytic reaction system for 20 min. From Figure S5, we could see the absorbance peak of oxTMB declined distinctly, which demonstrated that dissolved oxygen as an oxidant was an integral part of the catalytic oxidation system. As a result, we called the CdS QDs with photoswitchable enzyme-like activity photoswitchable oxidase mimetics.

**Figure 3:**
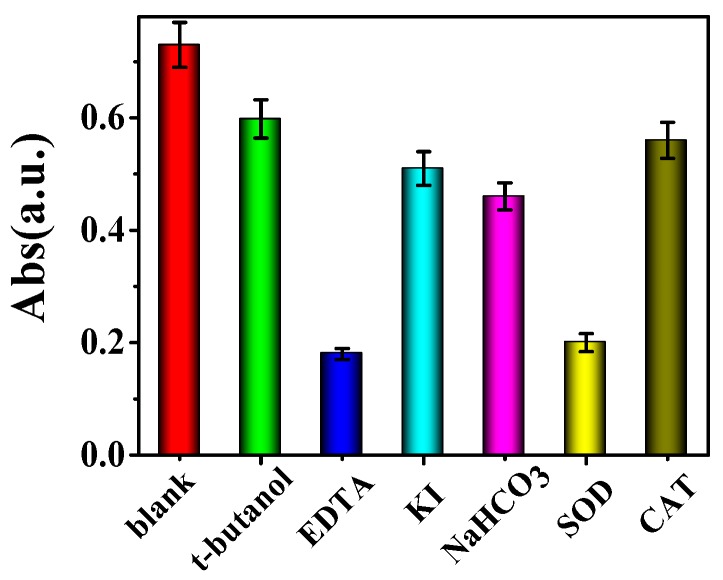
The effects of different scavengers on the oxidation of TMB under visible-light irradiation by PO_4_^3−^-capped CdS QDs.

For further clarifying the active species of the system responsible for the catalyzed oxidization, we applied a train of scavengers to capture the active species. As we know, KI and EDTA are scavengers of photo-generated holes (h^+^) [22], NaHCO_3_ and t-butanol are scavengers of hydroxyl radicals (^•^OH) [23], superoxide dismutase (SOD) is the scavenger of superoxide anions (O_2_^•−^)[24] and catalase (CAT) can catalyze the decomposition of H_2_O_2_ into water and oxygen [25]. As indicated in Figure 3, when these scavengers were introduced in our system, respectively, the absorbance of oxTMB declined in varying degrees. These experiments proved that the h^+^, ^•^OH, H_2_O_2_ and especially O_2_^•−^ were the reactive species responsible for TMB oxidation.

**Scheme 1:**
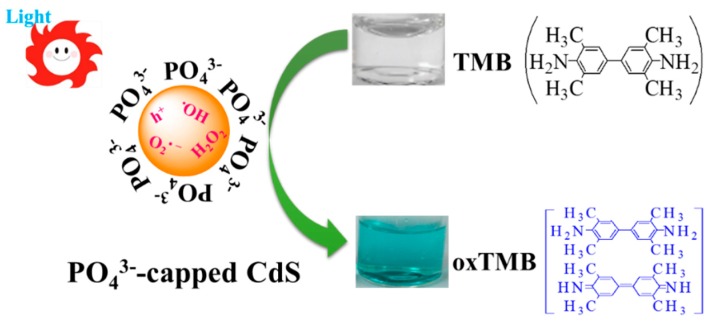
The mechanism of the photo-activated oxidase-like activity of PO_4_^3−^-capped CdS QDs.

We introduced photoelectrochemistry and electrochemistry experiments to confirm the reactive species generation mechanism of illuminated CdS QDs. Firstly, we investigated the photocurrent of PO_4_^3−^-capped CdS QDs in phosphate buffer. From Figure S6, we can see that the PO_4_^3−^-capped CdS QDs promptly generate a stable photocurrent with a reproducible response to on/off cycles, demonstrating the effective electron/hole generation and transfer of photoactivated PO_4_^3−^-capped CdS QDs. Subsequently, we employed linear sweep voltammetry (LSV) to study the conduction band (CB) (Figure S7A) and valence band (VB) (Figure S7B) edge of PO_4_^3−^-capped CdS QDs. The results showed that PO_4_^3−^-capped CdS QDs had a CB edge at −0.77 V and a VB edge at 0.93 V vs. Ag/AgCl (saturated KCl). That meant the CB and VB potentials of PO_4_^3−^-capped CdS QDs were −0.57 and 1.13 V vs. normal hydrogen electrodes (NHE), respectively. CdS is a favorable semiconductor material; under visible-light illumination, the electron of VB was excited into the CB and led to the VB producing h^+^. Because of the potential gradient between the CB (−0.57 V vs. NHE) of CdS QDs and the reduction potential of oxygen (0.815 V vs. NHE) [20], the oxygen in aqueous solution could accept the excited electrons and formed O_2_^•−^ or ^•^OH. The h^+^ were able to oxidize TMB directly because the VB potential of CdS QDs was above that of TMB with an oxidation potential in the range of 0.22–0.7 V [26]. The above experiments proved that O_2_^•−^, ^•^OH and h^+^ were the main reactive species responsible for TMB oxidation (Scheme 1).

### 2.3. Probing the Activity of Glucose Oxidase Using PO_4_^3−^-Capped CdS QDs

**Scheme 2:**
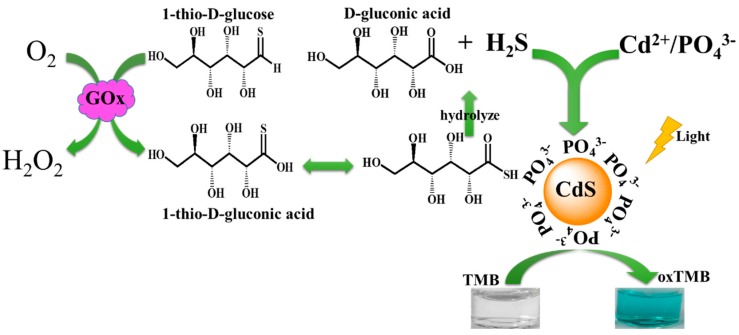
Detection of GOx activity through forming the photoswitchable oxidase mimetics of PO_4_^3−^-capped CdS QDs with the enzymatic product of GOx.

The coupling of biomolecules, for example natural enzymes, with nanomaterials formed in situ for target analytes has high sensitivity due to the low background signals, and it has received increasing attention by researchers [27,28]. As a biocatalyst, natural enzymes play an important role in the catalytic reaction, and even a very tiny amount of the enzyme can stimulate the occurrence of the catalytic reaction. PO_4_^3−^-capped CdS QDs formed in situ by GOx biocatalysis is an excellent mimetic oxidase that forms rapidly; once produced, it catalyzes the oxidation of TMB and further produces an amplified signal. Further, our detection does not require the use of intricate instruments, which makes it cheaper and more easy to operate. Based on the catalytic growth of PO_4_^3−^-capped CdS QDs in situ and the intrinsic oxidase-like property of PO_4_^3−^-capped CdS QDs, we developed a facile colorimetric method with efficient signal amplification to detect the enzymatic activity of GOx (Scheme 2).

For the sake of acquiring a better catalytic effect, we adjusted and controlled the reaction conditions of the GOx catalytic reaction, such as the reaction time and the concentration of 1-thio-β-d-glucose. As shown in Figure S8A, the enzyme catalytic reaction proceeded quickly and achieved a balance in 60 min. As can be seen from Figure S8B, we can see that with the increase of the 1-thio-β-d-glucose concentration, the absorbance of oxTMB gradually enhances, meaning that the catalytic effect is strengthened step by step. When the 1-thio-β-d-glucose concentration is greater than 1 mM, the absorbance of oxTMB hardly increases because of the depletion of GOx. Thus, we chose 60 min as the enzyme reaction time and 1 mM as the concentration of thio-β-d-glucose.

To confirm that the activity of GOx was the main factor in the production of CdS QDs, we verified the influence of the natural substrate β-d-glucose. Theoretically, the artificial substrate and the natural one were supposed to compete for binding with the active site of GOx. Thus, the presence of β-d-glucose consumes a part of the GOx and leads to the diminution of GOx to catalyze 1-thio-β-d-glucose. When the concentration of 1-thio-β-d-glucose is fixed, the increasing amount of β-d-glucose leads to a decreasing amount of its oxidative decomposition to H_2_S and gluconic acid and consequently weakens the catalytic effect of PO_4_^3−^-capped CdS QDs (Figure S8C). Based on the above results, we can see that the formation of PO_4_^3−^-capped CdS QDs in situ originated from the enzymatic reaction of 1-thio-β-d-glucose.

**Figure 4:**
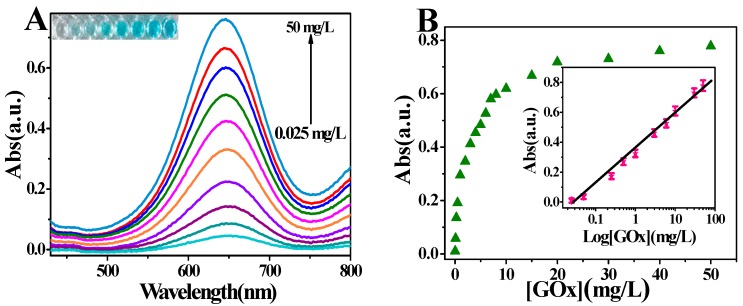
Analytical performances of the protocol for probing the activity of GOx. (**A**) UV/vis spectra of oxTMB catalyzed by the PO_4_^3−^-capped CdS QDs formed in situ under visible-light irradiation in the presence of different concentrations of GOx. From bottom to top, the concentrations of GOx are 0.025, 0.1, 0.5, 1.0, 2.0, 4.0, 6.0, 10.0, 20.0, 50.0 mg/L. The inset shows the corresponding color change of oxTMB; (**B**) Absorption change and the linear relationship (insert curve) between the absorbance of oxTMB at 652 nm and the concentration of GOx. The error bars indicate relative standard deviation of four repeated experiments.

To study the sensitivity of the proposed method for the detection of GOx activity, different amounts of commercially available GOx were added to the system and the absorption spectrum of oxTMB was recorded. As illustrated in Figure 4A, with the increase of the GOx concentration, the absorbance of oxTMB at 652 nm increased gradually and the absorbance increased linearly with the logarithmic enzymatic activity of GOx over the range from 25 μg/L to 50 mg/L with a detection limit of 6.6 μg/L (S/N = 3) (Figure 4B). The detection limit of this method for the enzymatic activity of GOx was comparable or even lower than that of other methods [15,29] reported, and it has a sufficiently wider linear range compared to other methods [30]. This colorimetric method for the detection of the enzymatic activity of GOx is convenient for detection with the naked eye and fairly inexpensive compared to other methods which are time-consuming or require specialized instruments.

## 3. Materials and Methods

### 3.1. Chemicals and Materials

The 1-thio-β-d-glucose was purchased from J&K (Shanghai, China). Glucose oxidase type VII (GOx), horseradish peroxidase (HRP), superoxide dismutase (SOD) from bovine liver, catalase (CAT), poly dimethyl diallyl ammonium chloride (PDDA, 20%, *w*/*w* in water, molecular weight = 200,000–350,000) and were purchased from Sigma-Aldrich, Co. (St. Louis, MO, USA). Na_2_S·9H_2_O was purchased from Shanghai Tongya Chemical Technology Co., Ltd (Shanghai, China). Glucose, ethylene diamine tetraacetic acid (EDTA), KI, *t*-butanol, 3,3′,5,5′-tetramethylbenzidine (TMB), 3CdSO_4_·8H_2_O, thioglycolic acid (TGA), chitosan (CS), Na_3_PO_4_ were purchased from Sinopharm Chemical Reagent Co., Ltd. (Shanghai, China). All other chemicals used were of analytical grade. All solutions were prepared with ultrapure water (18.2 MΩ·cm^−1^) obtained from a Healforce water purification system.

### 3.2. Instrumentation

High resolution transmission electron microscopy (HRTEM) images of PO_4_^3−^-capped CdS QDs were obtained on a JEOL JEM-2100 transmission electron microscope (Hitachi, Japan). The fluorescence spectra analysis and the resonance light scattering spectra were carried out on a Varian Cary Eclipse fluorescence spectrophotometer at room temperature. UV/Vis absorption spectroscopic measurements were carried out using a TU-1901 spectrophotometer (Beijing Purkinje General Instrument Co. Ltd., Beijing, China). A 300 W Xe lamp (NBeT, Beijing, China) equipped with an ultraviolet cutoff filter (λ ≥ 400 nm) was used as the irradiation source. Photoelectrochemical measurements were performed with a homemade photoelectrochemical system. Photocurrent was measured on a CHI 800C electrochemical workstation (Shanghai, China). PO_4_^3−^-capped CdS QDs modified ITO electrode was employed as the working electrode. A Pt wire was used as the counter electrode and a saturated Ag/AgCl as the reference electrode. All the photocurrent measurements were performed at a constant potential of 0 V (vs. saturated Ag/AgCl) in 0.2 M Na_2_SO_4_ solution as the supporting electrolyte. Linear sweep voltammetry (LSV) was used to determine the conduction/valence band edge of the PO_4_^3−^-capped CdS QDs, which was performed with a CHI 800C electrochemical workstation at room temperature under N_2_ atmosphere. A conventional three-electrode cell was used, including a Pt wire counter electrode, a saturated Ag/AgCl reference electrode. A glassy carbon electrode was used as working electrode. A 0.2 M Na_2_SO_4_ solution containing PO_4_^3−^-capped CdS QDs was used as the electrolyte solution. The detection of GOx activity by the absorption method was proceeded on 96 well plates by the microplate reader (SpectraMax M5, Sunnyvale, California, USA).

### 3.3. The Colorimetric Detection of Glucose Oxidase Activity

A certain amount of 1-thio-β-d-glucose were incubated with different amounts of GOx in citrate buffer (10 mM, pH 4.0) for 60 min at 37 °C. After that, 20 μL Cd^2+^/Na_3_PO_4_ mixture was added to the samples. Subsequently, the mixed solution was added with 20 μL of 5 mM TMB and then diluted to 200 μL by acetate buffer (200 mM, pH 4.0) and illuminated under visible light irradiation (λ ≥ 400 nm) for 10 min to allow development of the blue color, and the absorbance of the oxidized TMB (oxTMB) at 652 nm was measured.

## 4. Conclusions

In this work, we introduced a new, amplified approach for probing the activity of GOx based on coupling the enzymatic reaction of GOx and its in situ generation of PO_4_^3−^-capped CdS QDs with photoswitchable oxidase-mimicking activity. Under visible-light (λ ≥ 400 nm) stimulation, the generated PO_4_^3−^-capped CdS QDs were found to possess intrinsic oxidase-like activity which could catalyze the oxidation of the typical substrate (TMB) with dissolved oxygen acting as the electron acceptor. Kinetic analysis proved that the catalysis was in accordance with the typical Michaelis–Menten kinetics and had a higher affinity for TMB than that of HRP. The catalytic mechanism investigations indicated that photo-generated holes (h^+^), hydroxyl radicals (^•^OH), and especially superoxide anions (O_2_^•−^) were the reactive species for the catalytic reaction. By taking advantage of the GOx-triggered growth of PO_4_^3−^-capped CdS in situ and its intrinsic oxidase-like property, a facile, sensitive and selective colorimetric method was developed to probe the activity of GOx. It is expected that the PO_4_^3−^-capped CdS QDs generated in situ by GOx with photo-stimulated enzyme-mimicking activity may find wide potential applications in the fields of catalysis, biochemistry and biotechnology.

## Supplementary Materials

The following are available online at http://www.mdpi.com/1420-3049/21/7/902/s1.

## Abbreviations

The following abbreviations are used in this manuscript:

GOx	glucose oxidase
CdS QDs	cadmium sulfide quantum dots
TMB	3,3′,5,5′-tetramethylbenzydine
NPs	Nanoparticles
h^+^	photo-generated holes
^•^OH	hydroxyl radical
O_2_^•−^	superoxide anion
HRP	horseradish peroxidase
SOD	superoxide dismutase
CAT	Catalase
PDDA	poly dimethyl diallyl ammonium chloride
TGA	thioglycolic acid
CS	Chitosan
