# Supplementary material for: In Situ Enzymatically Generated Photoswitchable Oxidase Mimetics and Their Application for Colorimetric Detection of Glucose Oxidase
Source: Molecules. 2016 Jul 9;21(7):902. doi: 10.3390/molecules21070902 (PMC6273347; doi:10.3390/molecules21070902)
Supplement: Supplementary file 1 [file molecules-21-00902-s001.docx]

Supplementary Materials: In situ Enzymatically Generated Photoswitchable Oxidase Mimetics
and Its Application for Colorimetric Detection of Glucose Oxidase

Gen-Xia Cao, Xiu-Ming Wu, Yu-Ming Dong, Zai-Jun Li and Guang-Li Wang

**Figure S1.** The oxidation of TMB by a: Cd^2+^ (balck line) or b: S^2−^ (red line) alone under visible light irradiation. Inset image is the corresponding colour of the solution.

|  |  |
| --- | --- |
|  |  |

**Figure S2.** The optimization of catalytic oxidation conditions for TMB by PO_4_^3−^-capped CdS QDs. The effect of (**A**) different stabilizers of CdS and (**B**) illumination time on the catalytic oxidation of oxTMB under visible light irradiation. Blank is CdS without any stabilizer. The relative catalytic activities of PO_4_^3−^–capped CdS QDs under visible light irradiation () and HRP using 5 mmol/L H_2_O_2_ as an oxidant () at different solution pH (**C**) and temperature (**D**). Reaction conditions: [TMB] =0.5 mM, [Cd^2+^] =1.5 mM, [S^2−^] =0.02 mM, [PO_4_^3−^] =0.1 mM, irradiation time: 12 min.

**Figure S3.** Absorbance of oxTMB at 652 nm versus time by PO_4_^3−^-capped CdS QDs under visible light irradiation (λ ≥ 400 nm) with different concentrations of TMB. Reaction conditions: [TMB] = 0.5 mM, [Cd^2+^] = 1.5 mM, [S^2−^] = 0.02 mM, [PO_4_^3−^] = 0.1 mM, pH = 4.0.

**Figure S4.** Steady-state kinetic assay of PO_4_^3−^-capped CdS QDs under visible light irradiation (λ ≥ 400 nm) using TMB as the substrate. Inset is the Lineweaver–Burk plot of the double reciprocal of the Michaelis–Menten equation.

**Figure S5.** The oxidation of TMB which after bubbling the solution with high purity nitrogen for twenty minutes (**a**) and conducted in air with dissolved oxygen (**b**). Inset image is the corresponding colour of the solution.

**Figure S6.** Photocurrent–time performances of PO_4_^3−^–capped CdS QDs modified electrodes under visible light irradiation (λ ≥ 400 nm).

|  |  |
| --- | --- |

**Figure S7.** Cathodic and anodic linear potential scan for determining the conduction band (CB) (**A**) and valence band edge (VB) (**B**) of the PO_4_^3−^–capped CdS QDs specimens. PO_4_^3−^–capped CdS QDs with the cathodic and anodic scan in the deaerated 0.2 mol/L Na_2_SO_4_ solution.

|  |  |
| --- | --- |
|  | |

**Figure S8.** The effect of reaction time of enzyme (**A**); the concentration of thioglucose (**B**) and glucose (**C**) on the catalytic oxidation of TMB by the PO_4_^3−^–capped CdS QDs under visible light irradiation. The system contained [GO_x_] = 100 mg/L, [Cd^2+^] = 1.5 mM, [PO_4_^3−^] = 0.1 mM. Additionally, (**C**) [1-thio-β-d-glucose] =1 mM. The error bars indicated relative standard deviation of four repeated experiments.
